# Dietary Sources of Nitrate and Nitrite and Associations with Blood Pressure and Other Cardiovascular Disease Risk Factors in a Representative United Kingdom Population

**DOI:** 10.1016/j.tjnut.2025.11.018

**Published:** 2025-11-24

**Authors:** Hayat S Alzahrani, Helen B McKenna, Ditte A Hobbs, Kim G Jackson, Julie A Lovegrove

**Affiliations:** Department of Food and Nutritional Sciences, Institute for Cardiovascular and Metabolic Research, Institute of Food, Nutrition and Health, Hugh Sinclair Unit of Human Nutrition, University of Reading, Reading, Berkshire, United Kingdom

**Keywords:** blood pressure, drinking water, inorganic nitrate, processed meat, vegetables

## Abstract

**Background:**

Dietary inorganic nitrate from vegetable sources has been shown to lower blood pressure (BP) and improve endothelial function. However, the impact of nitrate from different dietary sources on BP remains unclear.

**Objectives:**

The objective of this study was to determine the relationships between dietary nitrate and nitrite from vegetables (with drinking water) and processed meats with BP and other cardiovascular disease (CVD) risk markers in a representative United Kingdom population.

**Methods:**

Data from the cross-sectional National Diet and Nutrition Survey years 1‒8, adults (19‒64 y) were used. For the analysis, a database of nitrate and nitrite concentrations in vegetables, drinking water, processed meats, and composite dishes was developed. The population was stratified into quartiles of increasing total and daily nitrate or nitrite intakes from vegetables (including drinking water) and processed meats to determine the relationships with biomarkers of CVD risk (BP, lipid profile, C-reactive protein, anthropometric measures, and glycemic control) using an analysis of covariance.

**Results:**

This dataset included 3338 adults (59% female) with a mean age of 43 y (standard deviation 12). Across increasing quartiles of vegetable nitrate intake, systolic BP (SBP), diastolic BP, waist circumference, waist-to-hip ratio, and glycated hemoglobin were lower in Q3 (95‒130 mg/d nitrate) than in Q1 (3‒65 mg/d) (*P* ≤ 0.038). Participants in Q4 (>131 mg/d) had lower pulse pressure, glucose, C-reactive protein, and total cholesterol concentrations than Q1 (*P* ≤ 0.05). Similar beneficial associations on SBP and lipid profiles were also evident for dietary nitrite intake from vegetables (*P* ≤ 0.05). In contrast, there was no difference in CVD risk biomarkers across quartiles of nitrate intake from processed meats, with higher SBP in Q4 (1.8‒3 mg/d nitrate) than Q1 (0.1‒0.8 mg/d) (*P* = 0.010).

**Conclusions:**

These data suggest that the dietary source of dietary nitrate and nitrite may play an important role in determining the relationship with BP and other CVD risk biomarkers in a representative United Kingdom population.

This trial was registered at clinicaltrials.gov as NCT05178875.

## Introduction

Cardiovascular diseases (CVD) are the greatest cause of death worldwide [1], with elevated blood pressure (BP) an independent risk factor [2]. CVD risk factors can be mitigated, in part, by addressing BP management, modifying the diet [3], and adopting a healthier lifestyle [4]. In particular, diets high in fruits and vegetables are associated with lower BP and risk of coronary artery disease and stroke [5]. This is supported by findings of a systematic review and meta-analysis, which reported a significant BP reduction with greater compliance to the dietary approaches to stop hypertension diet [6], which is high in vegetables, among other components.

Dietary nitrates are found in roots (e.g., such as beetroot) and green leafy vegetables. High levels of consumption is becoming increasingly recognized as a potential dietary strategy to reduce BP and enhance cardiovascular health in both healthy [7] and individuals with hypertension [8]. Although vegetables are the main source of dietary nitrates, contributing 70%‒80% to the total nitrate consumed [9], drinking water, and to a lesser extent cured and processed meats, also contribute [10]. However, dietary nitrate and nitrite intake estimation is challenging and often inaccurate due to a general lack of available data on nitrate and nitrite content in commonly used dietary analysis software databases. Furthermore, it has been recently hypothesized that dietary nitrate from vegetables produces greater bioactive effects, which are thought to enhance the potency and bioactivity of dietary nitrate due to other components, such as polyphenols, that are consumed with nitrate [11]. In contrast, nitrites are added to cured and processed meats as a preservative and to enhance taste. The International Agency for Research on Cancer 2015, reviewed >800 research publications and concluded that eating 50 g/d of processed meats was associated with higher BP [12] and an 18% higher risk of colorectal cancer [13]. One possible mechanism is the production of N-nitroso compounds from nitrates and nitrites in processed meats, which are potentially carcinogenic [14]. However, the impact of nitrate from different foods on BP and other CVD risk factors remains unclear.

Findings from acute and short-term intervention studies, including some of our own, have shown BP-lowering effects following intake of nitrate-rich beetroot juice and beetroot-enriched bread in normotensive adults [7]. However, there is a paucity of data on long-term habitual intake of dietary nitrate on BP and CVD outcomes in a larger population [15]. Furthermore, to our knowledge, no studies have investigated the relationships between the daily consumption of dietary nitrate from different sources (vegetables, processed meats, and drinking water) on CVD risk factors in the United Kingdom population. To fill this knowledge gap, a retrospective data analysis was performed to accurately estimate dietary nitrate consumption from various sources and investigate the relationship with BP and other CVD risk factors in participants (aged 19‒64 y) from the United Kingdom National Diet and Nutrition Survey (NDNS). We hypothesized that higher consumption of nitrates from vegetables and drinking water is associated with lower BP and beneficial effects on CVD risk markers, whereas nitrite intakes from cured and processed meats have unfavorable relationships.

## Methods

### Study design

The dataset used for this study was obtained from the NDNS years 1‒8 (2008‒2016) [16]. The NDNS is a continuous cross-sectional observational survey that has been ongoing in the United Kingdom since 2008, which aims to gather information on food consumption, nutrient intake, and nutritional status within a representative sample of the population. The NDNS design has been previously described in detail and published elsewhere [16]. Briefly, individuals completed a 4-d food diary and attended an interview to discuss habitual dietary intake and gather information on, for example, socio-economic class. At a follow-up visit, volunteers had a blood sample collected, gave a 24-h urine sample, and completed a range of anthropometric and BP measurements, including weight, BMI (in kg/m^2^), waist and hip circumferences (WC and HC, respectively), systolic BP (SBP), diastolic BP (DBP), and pulse rate. Pulse pressure (PP) was calculated by subtracting DBP from SBP. Blood samples were analyzed for CVD risk biomarkers, including lipids [triacylglycerol, total cholesterol (TC), LDL cholesterol, and HDL cholesterol], C-reactive protein (CRP), glucose, and glycated hemoglobin (HbA1c). The NDNS was conducted according to the guidelines laid down in the Declaration of Helsinki, and ethical approval for all procedures was granted by local research ethics committees covering all areas in the survey. All participants gave informed consent. A favourable ethical opinion for conduct was gained from the National Health Service Ethics Committee (18/NS/0085 IRAS Project ID: 238212) and the University of Reading Research Ethics Committee (STUDY Number – 11/18) for the retrospective analysis and registered at clinicaltrials.gov (NCT05178875).

### Development of the dietary nitrate and nitrite database

Since data on the nitrate content of vegetables and other nitrate-rich foods were not available in the NDNS nutrient databank, a database of the nitrate and nitrite contents of vegetables, processed meats, and drinking water (the major United Kingdom dietary sources) was developed. In the first instance, the researchers (DAH and HBM) manually searched the NDNS food database to remove food items that did not contain nitrate or nitrite and identified existing databases of food nitrate and nitrite concentrations. For this analysis, the nitrate concentrations of vegetables were taken from a database created as part of a meta-analysis of 255 publications assessing nitrate content in 180 vegetables and 22 herbs from worldwide samples published in 2017 [17]. The nitrate content of each vegetable was converted to milligrams of nitrate or nitrite per gram of food. Nitrite values for processed meats (including cured meats) were taken from papers (with or without meta-analysis) reporting the nitrite content of processed meats [10,18]. The values from these papers were then averaged to create a final representative value for these nitrate and nitrite contents.

The recipes for standard dishes (composite foods) were sourced from McCance and Widdowson’s The Composition of Foods Collection [19] or McCance and Widdowson’s supplementary textbooks, namely Meat and Meat Dishes [20] and Vegetables and Vegetable Dishes [21]. For a more accurate estimation of dietary nitrate, the loss of nitrate content during processing and cooking was calculated. The average loss was set at 47.5% which represented the percentage loss of nitrate and nitrite from boiling, given in the European Food Safety Authority (EFSA) report 2008 [22], unless a specific and referenced value was taken from the literature, especially for processed meats [10]. The nitrate content of each vegetable and processed meat was converted to milligrams of nitrate or nitrite per gram of food. The dietary nitrate content was calculated by multiplying the food items in grams by the nitrate content (milligrams) per gram, including vegetables and meat within composite dishes.

To estimate the nitrate intake from drinking water of the NDNS participants, all the United Kingdom water authorities were contacted to request information on the nitrate and nitrite content of the drinking water over the time period of the NDNS survey (Supplemental Table 1). For water authorities that did not provide this information, the nitrate and nitrite concentrations were compiled from the Drinking Water Inspectorate Annual Reports for 2008‒2016. None of the water authorities exceeded the maximum nitrate of 50 mg/L. These data were collated and sent to the offices of the National Centre for Social Research, where staff members linked the water authority data (based on the participants’ postcodes and the date the diet diaries were completed) to the anonymized subject identification code. To protect the identities of NDNS participants living in the smaller water authority areas, their data were either combined with a larger adjoining water authority area or excluded completely from the dataset. Total nitrate and nitrite intakes from the local tap water were then calculated by multiplying each participant’s total water intake in liters (for drinking and making coffee, and tea) from the diet diary with the nitrate and nitrite concentration (milligrams per liter) obtained from the water authorities. Finally, the individual total nitrate and nitrite consumption for each participant was calculated by summing the nitrate and nitrite from vegetables, water, and processed meats consumed in the 4-d dietary collection period and expressed as total nitrate or nitrite in milligrams per day (mg/d). The total dietary nitrate and nitrite intake data were then matched to the subject ID codes by National Centre for Social Research employees, which allowed the relationship between these intakes and anthropometric and CVD biomarkers to be determined.

### Statistical analysis

Statistical analysis was completed using IBM SPSS version 24 software. Weighting factors were applied to ensure the data collected from different years of the NDNS survey were comparable and could be pooled for the statistical analysis. Data were checked for normality using Q-Q plots and transformed to log10 where necessary prior to stratification according to quartiles of daily nitrate and nitrite intake from all sources, representing diets with the lowest (Q1) and highest (Q4) intakes. Daily nitrate and nitrite intakes were then calculated separately based on dietary sources (i.e., vegetables, including drinking water, and processed meats) before being stratified according to quartiles as above. An analysis of covariance was then used to determine differences between the CVD risk factors, including BP, anthropometric measurements, blood lipids, glucose, and CRP across increasing quartiles of intake of nitrate and nitrite from each dietary source. Age, sex, dietary energy intake (MJ), and BMI were used as covariates. A Bonferroni post hoc test was used to detect differences between quartiles of nitrate and nitrite intake when a significant difference was identified by analysis of covariance. In Table 1, data are presented as unadjusted means ± SD, and in TABLE 2, TABLE 3, TABLE 4, TABLE 5 as unadjusted estimated marginal means ± SEM, and *P* < 0.05 was considered statistically significant.

**TABLE 1:** Baseline characteristics of the National Diet and Nutrition Survey participants^1^

Characteristics	Values
*n* (M/F)	3338 (1368/1970)
Age, y	43 ± 12
BMI, kg/m^2^	26.3 ± 7.1
Blood pressure, mmHg	
Systolic	123 ± 16
Diastolic	74 ± 11
Pulse pressure	70 ± 11
Waist circumference, cm	92 ± 15
Hip circumference, cm	106 ± 11
Waist-to-hip ratio	0.87 ± 0.09
Total cholesterol, mmol/L	5.90 ± 1.07
Triacylglycerol, mmol/L	1.31 ± 0.92
HDL cholesterol, mmol/L	1.45 ± 1.24
LDL cholesterol, mmol/L	3.09 ± 0.92
Total:HDL cholesterol ratio	3.73 ± 1.25
Glucose, mmol/L	5.24 ± 1.18
C-reactive protein mg/L	1.61 ± 5.11
Glycated hemoglobin, %	5.52 ± 0.70
Dietary nitrate intake, mg/d	
Total	159.6 ± 62.5
Vegetables and drinking water	79.5 ± 47.2
Processed meats	56.2 ± 33.4
Dietary nitrite intake, mg/d	
Total	2.05 ± 1.71
Vegetables and drinking water	0.12 ± 0.54
Processed meats	2.01 ± 1.69

Abbreviations: BMI, body mass index; HDL cholesterol, low-density lipoprotein cholesterol; M/F, male/female; SD, standard deviation.

1 Values represent unadjusted means ± SD.

**TABLE 2:** Blood pressure, anthropometric measures, and other cardiovascular disease risk factors in the National Diet and Nutrition Survey participants according to quartiles of total dietary nitrate^1^

	Q1, *n* = 831 (19‒113 mg/d)	Q2, *n* = 832 (114‒149 mg/d)	Q3, *n* = 832 (150‒192 mg/d)	Q4, *n* = 843 (193‒572 mg/d)	*P* value^2^
SBP, mmHg	122 ± 0.6^ab^	123 ± 0.5^b^	121 ± 0.5^a^	123 ± 0.5^ab^	0.041
DBP, mmHg	73 ± 0.6^ab^	74 ± 0.5^b^	72 ± 0.5 ^a^	73 ± 0.5^ab^	0.044
PP, mmHg	37 ± 0.9	39 ± 0.8	39 ± 0.8	39 ± 0.8	0.141
Pulse rate, bpm	71 ± 0.5^b^	70 ± 0.4^ab^	67 ± 0.4^a^	69 ± 0.4^a^	<0.001
WC, cm	91.4 ± 0.2^b^	90.8 ± 0.2^ab^	90.2 ± 0.2^a^	90.4 ± 0.2^a^	0.001
HC, cm	105 ± 0.2	105 ± 0.2	105 ± 0.2	105 ± 0.2	0.485
WHR	0.87 ± 0.02^b^	0.86 ± 0.02^ab^	0.86 ± 0.02^a^	0.86 ± 0.02^a^	0.002
TC, mmol/L	5.09 ± 0.04	4.97 ± 0.04	4.97 ± 0.04	5.01 ± 0.04	0.248
TAG, mmol/L	1.35 ± 0.03	1.24 ± 0.03	1.26 ± 0.03	1.23 ± 0.03	0.082
HDL cholesterol, mmol/L	1.45 ± 0.01	1.41 ± 0.01	1.44 ± 0.01	1.43 ± 0.01	0.239
LDL cholesterol, mmol/L	3.06 ± 0.04	3.05 ± 0.03	2.95 ± 0.03	3.05 ± 0.03	0.360
TC:HDL cholesterol ratio	3.74 ± 0.05	3.77 ± 0.04	3.65 ± 0.04	3.73 ± 0.04	0.334
Glucose, mmol/L	5.05 ± 0.03	5.16 ± 0.03	5.10 ± 0.03	5.11 ± 0.03	0.120
CRP, mg/L	2.19 ± 0.01	1.98 ± 0.01	1.99 ± 0.01	2.05 ± 0.01	0.089
HbA1c, %	5.45 ± 0.02	5.48 ± 0.02	5.44 ± 0.02	5.43 ± 0.02	0.389

Abbreviations: ANCOVA, analysis of covariance; BMI, body mass index; CRP, C-reactive protein; DBP, diastolic blood pressure; HbA1c, glycated hemoglobin; HC, hip circumference; HDL cholesterol, high-density lipoprotein cholesterol; LDL cholesterol, low-density lipoprotein cholesterol; PP, pulse pressure; Q1‒Q4, quartile; SBP, systolic blood pressure; SEM, standard error of the mean; TAG, triacylglycerol; TC, total cholesterol; WC, waist circumference; WHR, waist-to-hip ratio.

1 Values are unadjusted estimated marginal means ± SEM for *n* = 3338. Values shown for quartiles of total dietary nitrate consumption are the minimum and maximum milligrams consumed per day.

2 Data were analyzed using ANCOVA with age, sex, energy intake, and BMI as covariates, and weighting factors were used in the analysis. Different superscript letters within the same row indicate a significant difference between quartile groups. *P* < 0.05 was considered a threshold for statistical significance.

**TABLE 3:** Blood pressure, anthropometric measures, and other cardiovascular disease risk factors in the National Diet and Nutrition Survey participants according to quartiles of dietary nitrate from vegetables (including drinking water) and processed meats^1^.

	Vegetables and water nitrate (mg/d)					Processed meats nitrate (mg/d)				
	Q1, *n* = 831 (3‒65)	Q2, *n* = 832 (66‒94)	Q3, *n* = 832 (95‒130)	Q4, *n* = 843 (131‒450)	*P* value^2^	Q1, *n* = 831 (3‒32)	Q2, *n* = 832 (33‒52)	Q3, *n* = 832 (53‒76)	Q4, *n* = 843 (77‒372)	*P* value^2^
SBP, mmHg	123 ± 0.6^b^	122 ± 0.5^ab^	121 ± 0.5^a^	122 ± 0.5^ab^	0.041	124 ± 0.7	123 ± 0.6	123 ± 0.6	124 ± 0.7	0.515
DBP, mmHg	74 ± 0.5^b^	72 ± 0.5^ab^	71 ± 0.5^a^	73 ± 0.5^ab^	0.011	74 ± 0.5	74 ± 0.5	73 ± 0.5	73 ± 0.5	0.430
PP, mmHg	39 ± 0.8	38 ± 0.8	37 ± 0.8	39 ± 0.8	0.470	36 ± 1.0	39 ± 0.9	40 ± 1.0	39 ± 1.0	0.077
Pulse, bpm	71 ± 0.5^b^	69 ± 0.4^ab^	69 ± 0.4^ab^	68 ± 0.4^a^	0.008	71 ± 0.5^b^	70 ± 0.5^ab^	68 ± 0.5^a^	69 ± 0.5^ab^	0.001
WC, cm	91 ± 0.2^b^	90 ± 0.2^ab^	89 ± 0.2^a^	90 ± 0.2^ab^	0.001	91 ± 0.3	91 ± 0.3	91 ± 0.3	91 ± 0.3	0.291
HC, cm	105 ± 0.2	104 ± 0.2	105 ± 0.2	105 ± 0.2	0.160	105 ± 0.2^ab^	105 ± 0.2^b^	105 ± 0.2^ab^	106 ± 0.2^a^	0.016
WHR	0.87 ± 0.02^b^	0.86 ± 0.02^ab^	0.85 ± 0.02^a^	0.86 ± 0.02^ab^	0.002	0.86 ± 0.02^ab^	0.87 ± 0.02^b^	0.86 ± 0.02^a^	0.86 ± 0.03^a^	0.010
TC, mmol/L	5.30 ± 0.08^b^	5.24 ± 0.07^ab^	5.26 ± 0.08^ab^	5.00 ± 0.07^a^	0.046	5.12 ± 0.03	5.13 ± 0.03	5.16 ± 0.03	5.13 ± 0.03	0.520
TAG, mmol/L	1.08 ± 0.01	1.21 ± 0.09	1.09 ± 0.09	1.07 ± 0.09	0.650	1.14 ± 0.08	1.13 ± 0.08	1.12 ± 0.6	1.16 ± 0.7	0.630
HDL cholesterol, mmol/L	1.45 ± 0.17	1.42 ± 0.16	1.44 ± 0.4	1.42 ± 0.16	0.680	1.42 ± 0.01	1.45 ± 0.01	1.44 ± 0.01	1.47 ± 0.01	0.126
LDL cholesterol, mmol/L	3.16 ± 0.07	3.11 ± 0.07	3.14 ± 0.07	2.98 ± 0.06	0.220	3.13 ± 0.03	3.10 ± 0.03	3.15 ± 0.03	3.16 ± 0.03	0.200
TC:HDL cholesterol ratio	3.76 ± 1.3	3.79 ± 1.2	3.77 ± 1.1	3.72 ± 1.2	0.616	3.82 ± 0.04	3.77 ± 0.04	3.81 ± 0.04	3.65 ± 0.04	0.140
Glucose, mmol/L	5.19 ± 0.03^b^	5.11 ± 0.03^ab^	5.06 ± 0.06^ab^	5.04 ± 0.02^a^	0.027	5.16 ± 0.02	5.21 ± 0.02	5.19 ± 0.02	5.16 ± 0.02	0.460
CRP, mg/L	2.19 ± 0.14^b^	2.08 ± 0.13^a^	2.04 ± 0.14^a^	2.02 ± 0.13^a^	0.001	2.16 ± 0.01	2.14 ± 0.01	2.17 ± 0.01	2.25 ± 0.01	0.151
HbA1c, %	5.50 ± 0.02^b^	5.44 ± 0.02^ab^	5.40 ± 0.02^a^	5.45 ± 0.6^ab^	0.017	5.52 ± 0.01	5.51 ± 0.01	5.45 ± 0.01	5.48 ± 0.01	0.127

Abbreviations: ANCOVA, analysis of covariance; BMI, body mass index; CRP, C-reactive protein; DBP, diastolic blood pressure; HbA1c, glycated hemoglobin; HC, hip circumference; HDL cholesterol, high-density lipoprotein cholesterol; LDL cholesterol, low-density lipoprotein cholesterol; PP, pulse pressure; Q1‒Q4, quartile; SBP, systolic blood pressure; SEM, standard error of the mean; TAG, triacylglycerol; TC, total cholesterol; WC, waist circumference; WHR, waist-to-hip ratio.

1 Values are unadjusted estimated marginal means ± SEM for *n* = 3338. Values shown for quartiles of total dietary nitrate consumption are the minimum and maximum milligrams consumed per day.

2 Data were analyzed using ANCOVA with age, sex, energy intake, and BMI as covariates, and weighting factors were used in the analysis. Different superscript letters within the same row indicate a significant difference between quartile groups. *P* < 0.05 was considered a threshold for statistical significance.

**TABLE 4:** Blood pressure, anthropometric measures, and other cardiovascular disease risk factors in the National Diet and Nutrition Survey participants according to quartiles of total dietary nitrite intake^1^.

	Q1, *n* = 831 (0.1‒0.9 mg/d)	Q2, *n* = 832 (1‒1.8 mg/d)	Q3, *n* = 832 (1.9‒3.1 mg/d)	Q4, *n* = 843 (3.2‒15.9 mg/d)	*P* value^2^
SBP, mmHg	121 ± 0.6^b^	122 ± 0.6^ab^	124 ± 0.7^a^	124 ± 0.7^a^	<0.001
DBP, mmHg	73 ± 0.4	73 ± 0.4	74 ± 0.4	73 ± 0.4	0.174
PP, mmHg	38 ± 0.7	38 ± 0.8	37 ± 0.8	40 ± 0.7	0.062
Pulse rate, bpm	69 ± 0.5	69 ± 0.6	68 ± 0.7	67 ± 0.8	0.413
WC, cm	91 ± 0.2^b^	90 ± 0.3^ab^	89 ± 0.3^a^	90 ± 0.3^ab^	0.015
HC, cm	104 ± 0.2	105 ± 0.2	104 ± 0.2	105 ± 0.2	0.635
WHR	0.87 ± 0.02^b^	0.86 ± 0.02^ab^	0.85 ± 0.02^a^	0.85 ± 0.02^a^	0.027
TC, mmol/L	4.99 ± 0.01^ab^	5.01 ± 0.01^ab^	5.10 ± 0.01^b^	4.92 ± 0.01^a^	0.023
TAG, mmol/L	1.06 ± 0.03	1.08 ± 0.03	1.11 ± 0.03	1.05 ± 0.05	0.298
HDL cholesterol, mmol/L	1.40 ± 0.01^b^	1.41 ± 0.01^ab^	1.46 ± 0.01^a^	1.45 ± 0.01^ab^	0.023
LDL cholesterol, mmol/L	3.03 ± 0.03^ab^	3.05 ± 0.03^ab^	3.09 ± 0.02^b^	2.94 ± 0.04^a^	0.038
TC:HDL cholesterol ratio	3.82 ± 0.04^b^	3.73 ± 0.04^ab^	3.74 ± 0.04^ab^	3.58 ± 0.05^a^	0.008
Glucose, mmol/L	5.53 ± 0.02	5.45 ± 0.01	5.55 ± 0.02	5.45 ± 0.02	0.079
CRP, mg/L	2.33 ± 0.01^b^	1.97 ± 0.01^a^	2.03 ± 0.01^a^	1.81 ± 0.01^a^	<0.001
HbA1c, %	5.48 ± 0.02	5.44 ± 0.02	5.43 ± 0.02	5.42 ± 0.02	0.193

Abbreviations: ANCOVA, analysis of covariance; BMI, body mass index; CRP, C-reactive protein; DBP, diastolic blood pressure; HbA1c, glycated hemoglobin; HC, hip circumference; HDL cholesterol, high-density lipoprotein cholesterol; LDL cholesterol, low-density lipoprotein cholesterol; PP, pulse pressure; Q1‒Q4, quartile; SBP, systolic blood pressure; SEM, standard error of the mean; TAG, triacylglycerol; TC, total cholesterol; WC, waist circumference; WHR, waist-to-hip ratio.

1 Values are unadjusted estimated marginal means ± SEM for *n* = 3338. Values shown for quartiles of total dietary nitrite consumption are the minimum and maximum milligrams consumed per day.

2 Data were analyzed using ANCOVA with age, sex, energy intake, and BMI as covariates, and weighting factors were used in the analysis. Different superscript letters within the same row indicate a significant difference between quartile groups. *P* < 0.05 was considered a threshold for statistical significance.

**TABLE 5:** Blood pressure, anthropometric measures, and other cardiovascular disease risk factors in National Diet and Nutrition Survey participants according to quartiles of dietary nitrite intake from vegetables (including drinking water) and processed meats^1^

	Vegetables and water nitrite (mg/d)					Processed meat nitrite (mg/d)				
	Q1, *n* = 831 (0.1‒0.3)	Q2, *n* = 832 (0.4‒0.7)	Q3, *n* = 832 (0.8‒1.4)	Q4, *n* = 843 (1.5‒9.2)	*P* value^2^	Q1, *n* = 831 (0.1‒0.8)	Q2, *n* = 832 (1‒1.7)	Q3, *n* = 832 (1.8‒3)	Q4, *n* = 843 (3.1‒15.9)	*P* value^2^
SBP, mmHg	124 ± 0.5^b^	123 ± 0.5^ab^	123 ± 0.5^ab^	122 ± 0.5^a^	0.007	120 ± 0.7^a^	121 ± 0.9^ab^	121 ± 0.8^ab^	123 ± 0.3^b^	0.010
DBP, mmHg	72 ± 0.4	73 ± 0.5	72 ± 0.6	72 ± 0.6	0.570	73 ± 0.5	73 ± 0.7	73 ± 0.6	74 ± 0.2	0.130
PP, mmHg	38 ± 0.8	38 ± 0.8	37 ± 0.8	38 ± 0.8	0.220	37 ± 1.7	38 ± 1.5	38 ± 1.3	39 ± 1.2	0.890
Pulse, bpm	70 ± 0.5	69 ± 0.6	69 ± 0.7	68 ± 0.8	0.370	69 ± 0.6	71 ± 0.8	70 ± 0.7	69 ± 0.3	0.080
WC, cm	91 ± 0.2^b^	91 ± 0.2^ab^	90 ± 0.2^a^	91 ± 0.2^ab^	0.005	92 ± 0.2	92 ± 0.2	92 ± 0.2	92 ± 0.2	0.507
HC, cm	105 ± 0.1	105 ± 0.1	105 ± 0.1	105 ± 0.2	0.270	105 ± 0.1^b^	105 ± 0.1^ab^	105 ± 0.1^ab^	106 ± 0.2^a^	0.008
WHR	0.87 ± 0.02^b^	0.86 ± 0.02^a^	0.86 ± 0.02^a^	0.86 ± 0.02^ab^	0.001	0.87 ± 0.02	0.87 ± 0.02	0.87 ± 0.02	0.87 ± 0.02	0.725
TC, mmol/L	5.00 ± 0.03	5.04 ± 0.03	5.06 ± 0.03	4.93 ± 0.03	0.229	4.89 ± 0.05	5.08 ± 0.07	5.09 ± 0.06	5.05 ± 0.03	0.059
TAG, mmol/L	1.06 ± 0.08	1.09 ± 0.08	1.09 ± 0.08	1.05 ± 0.08	0.501	1.08 ± 0.08	1.14 ± 0.08	1.12 ± 0.08	1.10 ± 0.08	0.244
HDL cholesterol, mmol/L	1.40 ± 0.01^b^	1.42 ± 0.01^ab^	1.47 ± 0.01^a^	1.46 ± 0.01^ab^	0.020	1.42 ± 0.01	1.43 ± 0.01	1.46 ± 0.01	1.45 ± 0.01	0.060
LDL cholesterol, mmol/L	3.03 ± 0.03	3.07 ± 0.03	3.06 ± 0.03	2.95 ± 0.03	0.148	2.90 ± 0.04	3.03 ± 0.06	3.04 ± 0.05	3.05 ± 0.03	0.082
TC:HDL cholesterol ratio	3.87 ± 0.04^b^	3.71 ± 0.04^ab^	3.60 ± 0.04^ab^	3.59 ± 0.04^a^	0.017	3.81 ± 0.04	3.76 ± 0.04	3.75 ± 0.04	3.70 ± 0.04	0.058
Glucose, mmol/L	5.18 ± 0.02	5.14 ± 0.02	5.11 ± 0.02	5.17 ± 0.02	0.267	5.18 ± 0.02	5.14 ± 0.02	5.15 ± 0.02	5.17 ± 0.02	0.389
CRP, mg/L	2.40 ± 0.01^b^	2.14 ± 0.02^a^	2.12 ± 0.02^a^	2.00 ± 0.01^a^	<0.001	2.20 ± 0.01	2.14 ± 0.02	2.12 ± 0.02	2.15 ± 0.01	0.823
HbA1c, %	5.55 ± 0.02^b^	5.48 ± 0.01^ab^	5.54 ± 0.02^ab^	5.45 ± 0.02^a^	0.028	5.50 ± 0.02	5.48 ± 0.01	5.47 ± 0.02	5.44 ± 0.02	0.094

Abbreviations: ANCOVA, analysis of covariance; BMI, body mass index; CRP, C-reactive protein; DBP, diastolic blood pressure; HbA1c, glycated hemoglobin; HC, hip circumference; HDL cholesterol, high-density lipoprotein cholesterol; LDL cholesterol, low-density lipoprotein cholesterol; PP, pulse pressure; Q1‒Q4, quartile; SBP, systolic blood pressure; SEM, standard error of the mean; TAG, triacylglycerol; TC, total cholesterol; WC, waist circumference; WHR, waist-to-hip ratio.

1 Values are unadjusted estimated marginal means ± SEM for *n* = 3338. Values shown for quartiles of total dietary nitrite consumptionare the minimum and maximum milligrams consumed per day.

2 Data were analyzed using ANCOVA with age, sex, energy intake, and BMI as covariates, and weighting factors were used in the analysis. Different superscript letters within the same row indicate a significant difference between quartile groups. *P* < 0.05 was considered a threshold for statistical significance.

## Results

This retrospective observational study investigated dietary nitrate and nitrite intake based on food diary records for 4745 adults aged 19‒64 y in years 1‒8 of the NDNS survey. Dietary data were sourced from *n* = 2697 from years 1‒4 (2008‒2012), *n* = 964 from years 5‒6 (2013‒2014), and *n* = 1081 from years 7‒8 (2015‒2016). After removing participants with incomplete anthropometric, BP, or blood biomarker outcome measures, 3338 subjects were included in this analysis, of which 59% were female. On average, the participants were middle-aged, with a BMI in the overweight range (Table 1).

### Dietary nitrate intake

Stratification of data according to quartiles of total dietary nitrate revealed SBP to be on average 2 mmHg lower in Q3 (150‒192 mg/d nitrate) than in Q1 (<113 mg/d nitrate; *P* = 0.041) and pulse rate to be slower in Q3 and Q4 than in Q1 (*P* < 0.001). WC and the waist-to-hip ratio were also lower in Q3 and Q4 than in Q1 (*P* = 0.027), with no differences in the blood CVD risk biomarkers between quartile groups (Table 2).

Similar findings were observed across increasing intakes of nitrate from vegetables and drinking water (Table 3). Participants in Q3 (95‒130 mg/d nitrate) had lower SBP (on average, ‒2 mmHg) and DBP (‒3 mmHg) than in Q1 (<65 mg/d nitrate) (*P* = 0.041 and *P* = 0.011, respectively). Pulse rate was also significantly slower in Q4 (>130 mg/d nitrate) than in Q1 (*P* = 0.008). Additionally, WC (*P* = 0.001), waist-to-hip ratio (*P* = 0.002) and HbA1c were lower in Q3 than in Q1 (*P* = 0.017). TC (*P* = 0.046), glucose (*P* = 0.027), and CRP (*P* = 0.001) concentrations were lower in Q4 than in Q1, with no differences found in other biomarkers between quartile groups.

For nitrate intake from processed meats, differences were not found in any of the measures of BP (SBP, DBP, or PP) across quartile groups. However, pulse rate was slower in Q3 than in Q1 (*P* = 0.001). HC was higher in Q4 (>77 mg/d nitrate) than in Q2 (33‒52 mg/d nitrate) (*P* = 0.016), resulting in a lower waist-to-hip ratio in Q4 than Q2 (*P* = 0.003). No significant differences were observed between quartiles of nitrate intake from processed meats for any of the circulating CVD risk biomarkers (Table 3).

### Dietary nitrite

Across quartiles of increasing total nitrite intake, SBP was on average 3 mmHg higher in Q3 (2‒3 mg/d nitrite) and Q4 (3‒15 mg/d nitrite) than in Q1 (<1 mg/d nitrite; *P* < 0.0001), with no significant changes observed in DBP, PP, or pulse rate. WC and waist-to-hip ratios were both lower in Q3 than in Q1 (*P* = 0.015 and *P* = 0.027, respectively). For the CVD risk biomarkers, TC (*P* = 0.023) and LDL cholesterol (*P* = 0.038) were lower in Q4 than in Q3, whereas HDL cholesterol was higher in Q3 than in Q1 (*P* = 0.008). CRP was significantly lower among all quartiles than Q1 (*P* < 0.0001) for all (Table 4).

In general, nitrites from vegetables and water were associated with more beneficial CVD risk marker profiles than nitrites from processed meat (Table 5). Participants in Q4 (1.5‒9.2 mg/d nitrite) had, on average, a 2 mmHg lower SBP than those in Q1 (>0.3 mg/d nitrite, *P* = 0.007). Nitrite intake from vegetables and water was also associated with lower WC in Q3 than in Q1 (*P* = 0.005) and a lower waist-to-hip ratio in both Q2 and Q3 than in Q1 for all (*P* < 0.001). Moreover, HDL cholesterol was higher in Q3 than in Q1 (*P* = 0.020), and there was a lower TC to HDL cholesterol ratio and HbA1c in Q4 than in Q1 (*P* = 0.028). CRP concentrations were lower in Q2, Q3, and Q4 than in Q1 (*P* < 0.001).

Higher nitrite intakes from processed meats were associated with a significantly greater SBP (on average 3 mmHg) in Q4 (1.8‒3 mg/d nitrite) than in Q1 (0.1‒0.8 mg/d nitrite) (*P* = 0.01). Furthermore, HC were higher in quartile Q4 than in Q2 (*P* = 0.008). No differences were observed in any of the other CVD risk biomarkers.

## Discussion

Using our collated database of the nitrate and nitrate contents of foods and drinking water, moderate intakes of nitrate from vegetables and drinking water had beneficial associations with BP and other CVD risk markers compared with the lowest intakes in a representative United Kingdom population. Interestingly, few associations were evident with nitrate and nitrite intakes from processed meats, with only nitrite intakes positively related to SBP. These results suggest that nitrate and nitrite from different dietary sources may have differential associations with CVD risk markers, likely influenced by accompanying nutrients (e.g., vitamin C and polyphenols) and food matrix (e.g., whole vegetables compared with juice) effects [23].

In agreement with the previous findings from observational studies reporting nitrate from vegetable sources only [24,25], we observed moderate to higher intakes of nitrate, 95‒130 mg/d [equivalent to just over a portion (80 g) of beetroot or spinach] compared with <65 mg/d (less than half a portion) from vegetables and water sources to be associated with a clinically relevant 2 mmHg lower SBP on a population level. Meta-regression analyses have reported relative risk reductions proportional to the magnitude of the SBP reductions, with every 10 mmHg reduction decreasing the risk of major CVD events by 20% and stroke by 27% [26]. Our analysis supported the findings from the Danish Diet, Cancer and Health study which reported that participants with the highest nitrate intake (median 141 mg/d) had lower SBP (−2.58 mmHg) and DBP (−1.38 mmHg) compared to those with the lowest intake, with the benefits reaching a plateau at moderate intake [27]. Similarly, finding from prospective cohort studies also confirmed this inverse relationship with BP and CVD risk, with the greatest risk reductions at moderate but not higher intakes of dietary nitrate, indicating a ceiling effect [15,25]. These findings suggest that small increases in inorganic nitrate, such as nitrate-rich vegetables and water, could help to lower BP. However, some differential associations have been reported between nitrate found in vegetables and water with CVD risk, with water-borne nitrate, even below regulatory limits (1.4–5.1 mg/L), being linked with a higher mortality risk [28]. These findings align with the biological rationale that vegetables provide co-nutrients that mitigate N-nitroso compound formation, unlike water.

In contrast to these studies demonstrating a beneficial effect of vegetable nitrate on mortality [15,25,27], prospective cohort studies have found that nitrite and nitrate intake from processed meats contributed to the increase of DBP by 3.1 mmHg in meat consumers who had enrolled in the Hellenic national nutrition and health survey [12]. Furthermore, other prospective cohort studies have found that both processed meats were associated with a higher hazard ratio of all-cause mortality due to the high nitrate and nitrite concentrations [29], and some forms of cancer and other non-communicable diseases [9]. A meta-analysis by Micha et al. [30] also reported that processed meat intake was associated with a 42% higher risk of coronary artery disease and a 19% increased risk of diabetes. In addition, vegetarians have been reported to have a 24% lower risk of death due to ischemic heart disease and a 20% lower risk of death from any cause than occasional meat eaters [31]. Although we observed a lack of association between nitrate intake from processed meats on CVD risk markers, including BP, lipid profile, and glycemia, processed meat nitrite intake was positively related to SBP. These data suggest that nitrite in processed meats may be one of the potential mediators of its detrimental association with CVD risk markers, although other components (e.g., salt) in this food cannot be discounted.

Studies have proposed potential mechanisms by which nitrate-rich foods and drinking water have a beneficial impact on cardiovascular health, such as improved vascular function, reduced inflammation [32], and an improved lipid profile [33]. After consumption, dietary nitrate is re-secreted via the salivary glands and converted to nitrite by nitrate-reducing bacteria within the oral microbiome. Nitrite is then converted to nitric oxide (NO) within the stomach or circulation, and acts as an effective vasodilator, resulting in acute and chronic BP reduction [34,35]. Previous intervention studies by our group and others have shown that acute [7,36] and chronic consumption of beetroot reduces BP [36,37] and improves vascular function [38]. A further study by Sobko et al. [39] compared the Japanese diet rich in vegetables with a Western-type diet in 25 healthy volunteers for 10 d. The naturally high nitrate content of the traditional Japanese diet (18.8 mg nitrate/kg body weight per day) was associated with a clinically significant lower DBP (4.5 mmHg) compared with the Western-type diet. Continuous monitoring of BP in acute settings has demonstrated a marked reduction in both SBP (10 mmHg) and DBP (8 mmHg) 3 h after consuming high nitrate beetroot juice, which was correlated with the greatest plasma nitrite concentrations [36]. Of note, these BP-lowering effects persisted 24 h after a single dose of inorganic dietary nitrate, demonstrating the potency of nitrate-rich vegetables on BP [36]. Furthermore, the postprandial impact of consuming 100 g, 250 g, and 500 g of beetroot juice containing 142 mg (moderate intake in this study), 353 mg, 707 mg dietary nitrate respectively on BP has also been shown to be dose dependent with a linear reduction in SBP compared with the low nitrate control, whereas DBP reached a plateau after 100 g beetroot juice [7]. In addition to this direct mechanism of BP lowering, dietary nitrate has been proposed to act indirectly through the interactions with other food components either within the food or consumed as part of the same meal (such as polyphenols, alcohol, and unsaturated fatty acids), with the formation of a variety of bioactive compounds [36,40]. An example is ethyl nitrite (produced in the stomach from dietary nitrate and alcohol), a potent smooth muscle relaxant, which may have a vasodilatory role in the cardiovascular system [40]. Furthermore, the reaction of NO with unsaturated fatty acids, such as linoleic acid, can produce nitroalkenes, which may support multiple-cell signaling events such as vasodilation, platelet aggregation, and reduced inflammation [41,42]. In the current study, we observed that individuals with the lowest dietary nitrate intakes from water and vegetables had a higher CRP (a marker for inflammation and a key CVD risk marker), compared to those with the highest intakes. These findings support those of a systematic review, which found dietary patterns characterized by higher factor loadings of green leafy vegetables to be related to lower CRP [40]. There is now increasing evidence that nitrate-rich plant foods and other sources of dietary nitrate can promote homeostasis of the immune system by counteracting inflammation and modulating immune cell phenotypes and function [30].

NO is becoming increasingly recognized as an important signaling molecule in the regulation of cardiometabolic health [41]. In the current study, we observed that individuals with lower intakes of dietary nitrate from water and vegetables had higher WC and waist-to-hip ratio, markers of glycemic control (plasma glucose and blood HbA1c), and plasma TC. Our findings agree with a cohort study that reported a higher urinary nitrate concentration (used as a biomarker of nitrate intake) in patients with lower abdominal obesity [42]. The authors speculated that modulation of adipose tissue function by dietary inorganic nitrate could impact body energy balance. Abdominal obesity is directly related to glycemic control and lipid regulation, with studies in control mice compared with those deficient in endothelial NO synthase (which develop a metabolic syndrome-like phenotype) demonstrating lower body weight and amount of visceral fat after consuming dietary nitrate [43]. These changes in body composition in the mice were associated with improved glucose tolerance and HbA1c concentrations at a nitrate dose that corresponded to a high intake of vegetables in a human diet (1 mM). In the current study, moderate to high nitrate intakes (95‒450 mg/d from vegetables and water) were associated with lower plasma glucose and HbA1c, respectively, compared with lower intakes. However, the limited data on relationships between dietary nitrate and glycemic control in humans warrant further research.

Studies in animal models of the metabolic syndrome have shown supplementation with spinach nitrate for 28 d to attenuate the elevation of triacylglycerol, total, and LDL cholesterol found in response to high-fructose and high-fat diets [33]. The beneficial effects of dietary nitrate on lipid homeostasis were found to be associated with a lower hepatic fat deposition and favorable effects on the F2-isoprostane concentrations, a marker of oxidative stress and lipid peroxidation. In our study, we found diets with moderate nitrate intake to be associated with lower plasma TC only; the mechanisms of which are unclear but could be related to the improvement in cellular metabolic homeostasis (fatty acid synthesis and oxidation and glucose uptake) mediated by NO or other nitrate-derived bioactive nitrogen oxides [41].

A strength of this study was the use of data from the NDNS, which ensured that the population studied was representative of the typical United Kingdom population. Furthermore, the development of a database of nitrate and nitrite from different dietary sources, including the main nutritional contributors: vegetables, drinking water, and processed meats, could be considered another strength. In particular, this database enabled a more representative assessment of nitrate and nitrite intakes from these combined dietary sources, and was the first to include nitrate and nitrite data from all water authority areas in the United Kingdom. Due to possible differences in the directions of the associations between nitrate derived from vegetables and drinking water, future studies could consider investigating these dietary sources separately [[44], [45], [46], [47], [48], [49]]. To estimate losses in cooking, a single cooking-retention factor was applied, but this did not account for losses due to washing or specific cooking methods [[49], [50], [51]]. This may have inadvertently led to exposure misclassification. This study also has some limitations. First, dietary nitrate and nitrite values were primarily derived from published literature and established food composition databases, which used mean values to estimate nitrate content in food. This approach may not fully account for the variability or influence of extreme values. Our database was compiled and the analysis performed prior to the publication of food composition databases for plant- [52] and animal-based foods [53] and demonstration of varying cooking methods on nitrate retention in 2022 [52]. In addition, vegetables are the major source of dietary nitrate, but we cannot discount the limited contribution from other foods, such as some fruits. Dietary data were obtained using the validated methodology (4-d diet diaries), which is widely accepted for population-level assessments in the United Kingdom, yet may not fully reflect long-term habitual intake. Also, this study was cross-sectional in design, which does not prove causation. Participants were generally healthy, so data may not be representative of those with CVD or other comorbidities. Although confounding factors were included in the statistical analysis, other factors could have affected BP and blood biomarkers, including other dietary components and physical activity.

In conclusion, this study shows that moderate habitual nitrate and nitrite intake from vegetables and water is associated with lower BP and other CVD risk markers. However, only nitrite from processed meats is associated with higher SBP. These findings suggest that diets containing moderate amounts of dietary nitrates could support BP control and CVD risk management, but that consuming high amounts of nitrite-rich processed meats may have opposing effects. Data such as these could be used to inform more specific population recommendations on the types of vegetable consumption.

## Author contributions

The authors’ responsibilities were as follows – JAL, KGJ, DAH, HSA: designed the research; HSA, HBM, DAH: conducted the research and analyzed the data, and drafted the paper; KGJ, JAL: provided feedback and guidance on previous drafts of this paper and were responsible for final content; and all authors: read and approved the final manuscript.

## Data availability

Data described in the manuscript can be freely accessed from the United Kingdom data services website. The datasets used and analyzed are available from the corresponding author on reasonable request.

## Funding

The PhD studentship awarded to HSA by the King Saud University, Riyadh, Saudi Arabia supported this research. The Ongoing Research Funding Program, (ORF-2025-1127), from the King Saud University, Riyadh, Saudi Arabia, supported HSA during the drafting of the manuscript

## Conflict of interest

JAL is Deputy Chair of the United Kingdom Scientific Advisory Committee of Nutrition (SACN). The views expressed in the manuscript are those of the author and do not necessarily represent those of SACN or the Department of Health and Social Care. All other authors report no conflicts of interest.
