# Supplementary material for: Dietary Sources of Nitrate and Nitrite and Associations with Blood Pressure and Other Cardiovascular Disease Risk Factors in a Representative United Kingdom Population
Source: J Nutr. 2025 Nov 24;156(2):101249. doi: 10.1016/j.tjnut.2025.11.018 (PMC12975364; doi:10.1016/j.tjnut.2025.11.018)
Supplement: Multimedia component 1 [file mmc1.docx]

**Supplemental Table 1**: List of the UK Water Authorities.

| Water Authority | Location |
| --- | --- |
| Albion Water | Herts |
| Independent Water Networks | Cardiff |
| SSE Water (Scottish and Southern Energy plc) | Scotland |
| Peel Water Networks | The Trafford Centre |
| Veolia Water | London |
| Northern Ireland water | Northern Ireland |
| Affinity Water | Essex |
| Affinity Water | Kent |
| Anglian Water Services Ltd | Huntingdon |
| Bristol Water plc | Bristol |
| Cholderton & District Water Company Ltd | Wiltshire |
| United Utilities Water plc | Warrington |
| Northumbrian Water Ltd | Durham |
| Portsmouth Water plc | Hants |
| Sembcorp Bournemouth Water Ltd | Bournemouth |
| Dee Valley Water plc | North Wales |
| Northumbrian Water Ltd | Durham |
| Dwr Cymru Welsh Water | Welsh |
| Sembcorp Bournemouth Water Ltd | Bournemouth |
| Severn Trent Water Ltd | Coventry |
| South East Water Ltd | Kent |
| Southern Water Services Ltd | Sussex |
| South Staffordshire Water plc | Cambridge |
| South West Water Ltd | Exeter |
| Sutton & East Surrey Water plc | Surrey |
| Thames Water Utilities Ltd | Reading |
| Wessex Water Services Ltd | Bath |
| Yorkshire Water Services Ltd | Bradford |
| Severn Trent Water Ltd | Coventry |
